# Global prevalence of mild cognitive impairment among older adults living in nursing homes: a meta-analysis and systematic review of epidemiological surveys

**DOI:** 10.1038/s41398-023-02361-1

**Published:** 2023-03-11

**Authors:** Pan Chen, Hong Cai, Wei Bai, Zhaohui Su, Yi-Lang Tang, Gabor S. Ungvari, Chee H. Ng, Qinge Zhang, Yu-Tao Xiang

**Affiliations:** 1 Unit of Psychiatry, Department of Public Health and Medicinal Administration, & Institute of Translational Medicine, Faculty of Health Sciences, University of Macau, Macao, Macao SAR China; 2 Centre for Cognitive and Brain Sciences, University of Macau, Macao, Macao SAR China; 3 Institute of Advanced Studies in Humanities and Social Sciences, University of Macau, Macao, SAR China; 4 School of Public Health, Southeast University, Nanjing, China; 5 Department of Psychiatry and Behavioral Sciences, Emory University, Atlanta, GA USA; 6 Atlanta Veterans Affairs Medical Center, Decatur, GA USA; 7 Section of Psychiatry, University of Notre Dame Australia, Fremantle, Australia; 8 Division of Psychiatry, School of Medicine, University of Western Australia, Perth, Australia; 9 Department of Psychiatry, The Melbourne Clinic and St Vincent’s Hospital, University of Melbourne, Richmond, Victoria, Australia; 10 Beijing Key Laboratory of Mental Disorders, National Clinical Research Center for Mental Disorders, National Center for Mental Disorders, Beijing Anding Hospital, Capital Medical University & Advanced Innovation Center for Human Brain Protection, Capital Medical University, Beijing, China

**Keywords:** Psychiatric disorders, Scientific community

## Abstract

Mild cognitive impairment (MCI) is the early stage of cognitive impairment between the expected cognitive decline of normal aging and the more serious decline of dementia. This meta-analysis and systematic review explored the pooled global prevalence of MCI among older adults living in nursing homes and its relevant factors. The review protocol was registered in INPLASY (INPLASY202250098). PubMed, Web of Science, Embase, PsycINFO, and CINAHL databases were systematically searched from their respective inception dates to 8 January 2022. The inclusion criteria were made based on the PICOS acronym, as follows: Participants (*P*): Older adults living in nursing homes; Intervention (*I*): not applicable; Comparison (*C*): not applicable; Outcome (*O*): prevalence of MCI or the data can generate the prevalence of MCI according to study-defined criteria; Study design (S): cohort studies (only baseline data were extracted) and cross-sectional studies with accessible data published in a peer-reviewed journal. Studies involving mixed resources, reviews, systematic reviews, meta-analyses, case studies, and commentaries were excluded. Data analyses were performed using Stata Version 15.0. Random effects model was used to synthesize the overall prevalence of MCI. An 8-item instrument for epidemiological studies was used to assess the quality of included studies. A total of 53 articles were included involving 376,039 participants with a mean age ranging from 64.42 to 86.90 years from 17 countries. The pooled prevalence of MCI in older adults in nursing homes was 21.2% (95% CI: 18.7–23.6%). Subgroup and meta-regression analyses revealed that the screening tools used were significantly associated with MCI prevalence. Studies using the Montreal Cognitive Assessment (49.8%) had a higher prevalence of MCI than those using other instruments. No significant publication bias was found. Several limitations warrant attention in this study; for example, significant heterogeneity between studies remained and some factors associated with the prevalence of MCI were not examined due to insufficient data. Adequate screening measures and allocation of resources are needed to address the high global prevalence of MCI among older adults living in nursing homes.

## Introduction

Mild cognitive impairment (MCI) is often defined as complaints of memory deficits and abnormal memory function that differ from healthy age-matched individuals, normal general cognitive function, and activities of daily living, which, however, does not meet the criteria of dementia [1–3]. It may be a precursor of dementia, being a transitional state from normal aging to dementia. A previous study found that over 60% of people with MCI went on to develop clinical dementia during their life [4]. The conversion rate varied among different studies with an average annual rate of 10–15% [2, 5–8], and after 6 years over 80% developed dementia [4]. A meta-analysis found that the proportion of those with MCI who progressed to dementia was 39.2% in clinical settings such as memory clinics or hospitals, while the corresponding figure was 21.9% in community populations [9]. Another survey reported that individuals with MCI converted to probable dementia at a high-rate of 241.3/1,000 person-years (PY), which was almost four times the risk of those with normal cognition [10]. In addition, some studies suggested that participants with MCI had increased mortality compared to those with normal cognition [11–13].

Nursing homes are facilities for people who cannot be cared for at home but do not need to be in a hospital. They often provide a family-style environment with 24-h functional support and care for older people who need help with activities of daily living, have complex health care needs, and are more vulnerable [14]. Impaired cognitive function is one of the major contributing factors leading to the placement of older people in nursing homes [15]. For instance, a previous study found that mild to moderate cognitive impairment was associated with more than 7 times higher risk of nursing home admission and more than 5 times higher risk of death [16] than those without cognitive impairment. Nursing homes are a suitable choice to care for older people with increased severity of cognitive impairment as the professional care provided can improve their quality of life and alleviate the burden on family caregivers [17]. Epidemiological studies of MCI in those living in nursing homes provide a good basis to allocate sufficient health resources to provide early identification, prevention and timely treatment of MCI before it develops into dementia [18]. Studies that examined the prevalence of MCI among older adults living in nursing homes found mixed results ranging from 4.0% to 87.4% [18–20]. Further, most meta-analyses of the prevalence of MCI focused only on community-dwelling populations [10, 21–23]. For example, a meta-analysis of the overall prevalence of MCI reported a prevalence of 17.3% in community-dwelling older people [24]. Considering that the prevalence of MCI in those living in nursing homes appeared higher than that in the community [25, 26], the epidemiological findings obtained in the community could not be generalized to nursing home residents. To date, no meta-analysis or systematic review on the prevalence of MCI among older adults living in nursing homes has been published.

To fill in this gap, this meta-analysis examined the pooled global prevalence of MCI among older adults living in nursing homes and its associated factors.

## Methods

### Search strategy

This meta-analysis was conducted based on the guidelines of Meta-Analysis Of Observational Studies in Epidemiology (MOOSE) [27] and the Preferred Reporting Items for Systematic Reviews and Meta-Analyses (PRISMA) [28]. The registration number of this protocol was INPLASY202250098. A comprehensive literature search was conducted by two researchers (PC and HC) independently in major international databases from their inception dates to 8 January 2022, including PubMed, Web of Science, Embase, PsycINFO, and CINAHL. Search terms were as follows: (“cognitive dysfunction”[MeSH Terms] OR “mild cognitive impairment” or “MCI”) AND (“Nursing Homes” OR “Nursing Home” OR “Intermediate Care Facilities” OR “Intermediate Care Facility” OR “Skilled Nursing Facilities” OR “Skilled Nursing Facility” OR “Extended Care Facilities” OR “Extended Care Facility” OR “convalescence home” OR “convalescence hospital” OR “long-term care” OR “old age homes” OR “residential homes” OR “nursing home*” OR “residential care” OR “institutionalization*” OR “nursing home placement*” OR “nursing home admission*” OR “Homes, Nursing”) AND (“aged” OR “old age” OR “elderly” OR “late-life” OR “geriatric*” OR “older adult” OR “elder*”) AND (“prevalence” OR “epidemiology” OR “rate”). The search strategy is shown in Supplementary Table S1. The reference lists of relevant reviews [22, 29–31] were also searched manually for additional studies.

### Inclusion and exclusion criteria

**Fig. 1:**
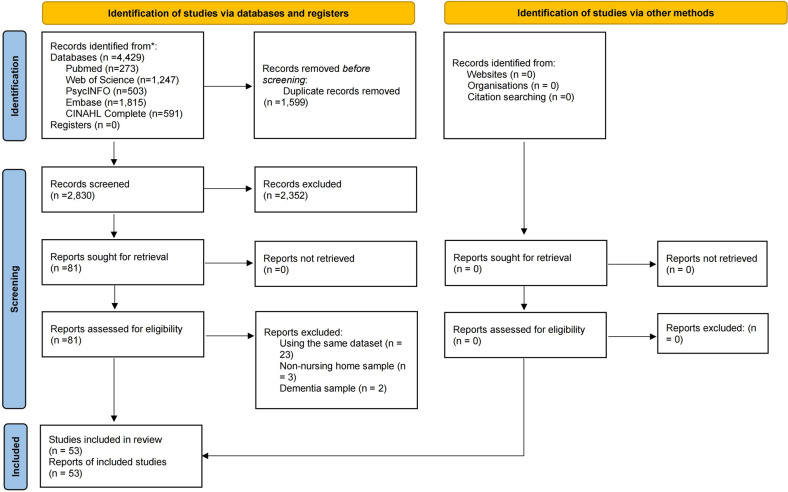
Flow chart of study selection.

The same two researchers independently screened the titles and abstracts of publications and then read the full texts of the relevant publications to identify eligible studies. The inclusion criteria were made based on the PICOS acronym, as follows: Participants (*P*): Older adults living in nursing homes; Intervention (*I*): not applicable; Comparison (*C*): not applicable; Outcome (*O*): prevalence of MCI or the data can generate the prevalence of MCI according to study-defined criteria; Study design (S): cohort studies (only baseline data were extracted) and cross-sectional studies with accessible data published in a peer-reviewed journal. Studies involving mixed resources (e.g., nursing homes and communities), reviews, systematic reviews, meta-analyses, case studies, and commentaries were excluded. When multiple studies based on the same dataset were published, only the one with the largest sample size was included. Any discrepancies in the above procedures were resolved by a discussion with a third investigator (YTX). The process of study selection is shown in Fig. 1.

### Data extraction and study quality assessment

Data were extracted independently by two investigators (PC and HC), including study characteristics (first author, publication year, survey time, countries, study design, sampling methods, and screening tool used for MCI) and sample characteristics (sample size, mean age, proportion of males, and number of participants with MCI). An 8-item instrument for epidemiological studies [32, 33] was used to assess the quality of included studies, including: (1) Target population was defined clearly; (2) Probability sampling or entire population surveyed; (3) Response rate was equal or greater than 80%; (4) Non-responders were clearly described; (5) Sample was representative of the target population; (6) Data collection methods was standardized; (7) Validated criteria were used to diagnose MCI; and (8) Prevalence estimates were given with confidence intervals and detailed by subgroups (if applicable). The total score ranges from 0 to 8, with low (0–3), moderate (4–6), and high (7–8) quality levels [34]. Disagreements between investigators in study assessments were resolved by a discussion with a third investigator (YTX).

### Statistical analysis

The meta-analysis was performed by Stata version 15 software. Due to different demographic data and methodology (e.g., sampling method) between the studies, the pooled prevalence of MCI and 95% confidence intervals (CIs) were calculated using a random-effects model [35]. Cochran’s Q test and *I*^2^ statistics were used to quantify the heterogeneity across studies, the *P* < 0.1 or *I*^2^ > 50% was defined as significantly high heterogeneity [36]. Subgroup analyses for categorical variables (study regions, countries by economic status according to the World Bank’s criteria [37], sampling method, scales on MCI, age group, and survey year) and meta-regression analysis for continuous variables (mean age, male proportion, and quality assessment score) were used to explore the sources of potential heterogeneity. Sensitivity analyses were performed to evaluate the stability of results by excluding each study, one by one. Begg’s funnel plot and Egger’s tests were used to assess the publication bias of the included studies. A *P* < 0.05 (two-tailed) was considered statistically significant.

## Result

### Characteristics of the studies

In total, 4429 relevant publications were searched from the databases. After removing 1599 duplicate records, 2830 titles and abstracts were screened and the full text of 81 publications were reviewed for eligibility. Of them, 28 were excluded due to overlapping data based on the same dataset (*n* = 23), non-nursing home samples (*n* = 3) and dementia samples (*n* = 2). Finally, 53 eligible studies were included in this meta-analysis. In total, 376,039 participants with a mean age ranging from 64.42 to 86.90 years from 17 countries were included. Most of the studies were conducted in Europe & Central Asia (29; 54.7%), followed by North America (14; 26.4%), East Asia & Pacific (8; 15.1%), Middle East & North Africa (1; 1.9%), and Sub-Saharan Africa (1; 1.9%). The survey years ranged from 1982 to 2019. Nearly four-fifths of the studies were cross-sectional (42; 79.2%) and more than half (31; 58.5%) used convenience sampling. Of the 13 MCI screening measures, the Mini-Mental State Examination (MMSE) (24; 45.3%) was the most frequently used tool. Study quality assessment scores ranged from 4 to 7; 48 (90.6%) were considered “moderate” quality and 5 (9.4%) were considered “high” quality. Detailed characteristics and quality assessment scores are presented in Table 1 and Supplementary Table S2.

**Table 1:** Sociodemographic characteristics of studies included in this systematic review and meta-analysis.

No.	First author	Country	Survey time	Study design	Sampling method	No. of subjects	Male (%)	Mean age (years)	Screening scale	No. MCI	Quality assessment score
1	Björk et al. [59]	Sweden	2013–2014	Cross-sectional	Random	4245	31.9	85.56	GCS	1067	5
2	Bo et al. [40]	Italy	2013	Cohort	NR	863	32.6	82.9	MMSE	168	4
3	Chun et al. [67]	USA	NR	Cross-sectional	Convenience	155	36.8	79.93	CAREDiag	58	5
4	Closs et al. [68]	UK	NR	Cross-sectional	Convenience	113	23.9	84.5	MMSE	24	5
5	Cocco et al. [69]	Italy	NR	Cross-sectional	Cluster	1976	NR	84.09	MMSE	138	6
6	Creighton et al. [70]	Australia	2015–2016	Cross-sectional	Random	178	NR	NR	MMSE	64	6
7	de Jong-Schmit et al. [71]	Norway	2014–2015	Cross-sectional	Convenience	412	24.2	86.9	MMSE	74	5
8	Díaz et al. [72]	Spain	NR	Cohort	Convenience	2849	31.7	85.21	MMSE	755	5
9	Garcia-Gollarte et al. [73]	Spain	2016–2017	Cohort	Convenience	531	24.7	86.7	SPMSQ	75	4
10	Gjøra et al. [61]	Norway	2017–2019	Cohort	Convenience	569	NR	NR	MoCA	70	5
11	Gruber-Baldini et al. [74]	USA	1997–1998	Cross-sectional	Random	2022	NR	NR	MDS Cognition Scale	586	5
12	Guliani et al. [75]	Canada	2004–2015	Cohort	Convenience	16,581	33.7	85	CPS	2883	6
13	Guo et al. [8]	China	2008–2009	Cross-sectional	Random	264	50.8	77.8	MMSE	35	7
14	Guthrie et al. [76]	Canada	2009–2014	Cross-sectional	Convenience	110,578	29.1	86.9	CPS	17,189	4
15	Hagglund et al. [77]	Sweden	NR	Cohort	Cluster	391	46.5	84	Medical record	47	6
16	Hasche et al. [78]	USA	2000–2003	Cohort	Convenience	551	22.9	72.4	SPMSQ	32	5
17	Hayajneh et al. [18]	Jordan	NR	Cross-sectional	Convenience	182	91.3	64.42	MoCA	159	5
18	Kijowska et al. [25]	Poland	2015	Cross-sectional	Random	1587	32.3	78.15	CPS	341	7
19	Kowalska et al. [79]	Poland	2007–2010	Cross-sectional	Convenience	254	19.3	77.7	MMSE	42	5
20	Lachs et al. [80]	USA	2009–2013	NR	Random	2011	27.5	84.14	CAREDiag	420	7
21	Lapane et al. [81]	USA	2011–2016	Cross-sectional	Convenience	180,780	25	NR	MDS 3.0 CFS	48,051	4
22	Lindbo et al. [82]	Sweden	2007 and 2013	Cross-sectional	Cluster	4397	32	84.7	GCS	1104	5
23	Lövheim et al. [83]	Sweden	1982 and 2000	Cross-sectional	Convenience	6864	NR	NR	GCS	3935	6
24	Lueken et al. [84]	Germany	NR	Cross-sectional	Convenience	356	17.1	85.6	MMSE	28	4
25	Malara et al. [85]	Italy	2010	Cross-sectional	NR	174	30	78.91	MMSE	12	4
26	Mansbach et al. [26]	USA	2012–2014	Cross-sectional	Convenience	477	NR	NR	BCAT	108	4
27	Manz et al. [86]	USA	NR	Cross-sectional	Random	100	26	83	SPMSQ	9	6
28	Margari et al. [87]	Italy	NR	Cross-sectional	Convenience	201	17.9	81.85	MMSE	63	4
29	McCusker et al. [41]	Canada	NR	Cross-sectional	Convenience	274	43.8	NR	MMSE	66	4
30	McDougall et al. [88]	USA	NR	Cross-sectional	Convenience	30	26.7	73	MMSE	10	4
31	Namasivayam-MacDonald et al. [89]	Canada	2015–2016	Cross-sectional	Random	622	31.7	86.8	CPS	75	4
32	Netten et al. [90]	UK	1995–1996	Cross-sectional	Convenience	16,172	NR	NR	CPS	2264	7
33	Onishi et al. [91]	Japan	2005–2006	NR	Random	70	35.7	NR	CPS	10	5
34	Parmelee et al. [92]	USA	1985–1991	Cross-sectional	Convenience	758	30	83.3	Blessed test	297	7
35	Ramlall et al. [56]	South Africa	NR	Cross-sectional	Random	140	30.7	75.2	MMSE	38	5
36	Redaelli et al. [93]	Italy	NR	Cross-sectional	Convenience	378	21.2	85.96	MMSE	56	5
37	Rodríguez-Rejón et al. [94]	Spain	2013–2016	Cross-sectional	Random	249	25	84.9	Pfeiffer test	31	4
38	Seijo-Martinez et al. [95]	Spain	NR	Cross-sectional	Convenience	1167	35.6	81.44	MMSE	408	4
39	Sjölund et al. [96]	Sweden	2012	Cross-sectional	Random	213	31.5	85.4	MMSE	50	4
40	Skoldunger et al. [97]	Sweden	2013–2014	Cross-sectional	Random	4,831	32.2	85.5	GCS	1067	5
41	Steenbeek et al. [98]	Netherlands	2013–2019	Cohort	Convenience	1,256	34.6	83.2	CPS	247	4
42	Sutcliffe et al. [99]	UK	NR	Cross-sectional	Convenience	308	31.2	82.8	MMSE	64	4
43	Thompson et al. [100]	Canada	2007–2012	Cross-sectional	Random	962	NR	NR	CPS	73	6
44	Vincze et al. [62]	Hungary	NR	NR	NR	2142	NR	NR	MMSE	386	4
45	Volicer et al. [101]	Netherland	2008–2009	Cross-sectional	Convenience	1851	29.2	83.6	CPS	183	6
46	Wang et al. [102]	China	2019	Cross-sectional	Convenience	1026	NR	NR	MMSE	206	4
47	Wongpakaran et al. [103]	Thailand	2010	Cross-sectional	Convenience	81	44.4	76.96	MMSE	14	5
48	Wulff et al. [104]	Germany	NR	Cross-sectional	Random	560	39	81.2	MMSE	82	4
49	Xu et al. [20]	China	2017–2018	Cross-sectional	Convenience	1087	56.5	77.5	MMSE	42	4
50	Xu et al. [105]	China	NR	Cross-sectional	Convenience	943	35.8	84	MMSE	195	6
51	Yang et al. [60]	China	2014	Cross-sectional	Random	908	35.3	84	MMSE	196	4
52	Zuluaga et al. [106]	Norway	NR	Cross-sectional	Convenience	135	NR	85.7	Pfeiffer test	16	4
53	Zuluaga et al. [107]	Spain	2009	Cross-sectional	Convenience	215	27.1	82.9	Pfeiffer test	70	4

*GCS* Gottfries cognitive scale, *MMSE* Mini-Mental State Examination, *CAREDiag* The Care Dementia Diagnostic Scale, *SPSMQ* Short Portable Mental Status Questionnaire, *BCAT* Brief Cognitive Assessment Tool, *MoCA* Montreal Cognitive Assessment, *MDS Cognition Scale* Minimum Data Set-Cognition Scale, *CFS* MDS 3.0-CFS Cognitive Function Scale, *CPS* Minimum Data Set 2.0-Cognitive Performance Scale, *NR* not report.

### Prevalence of mild cognitive impairment

**Fig. 2:**
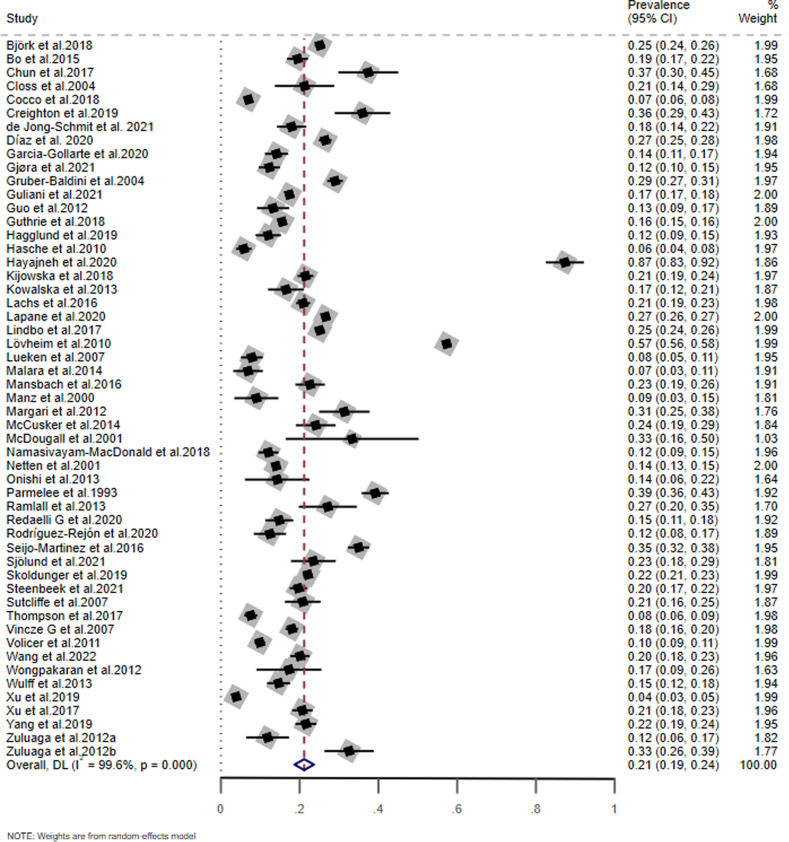
The prevalence of MCI among older adults living in nursing homes. Pooled prevalence was estimated by random-effects model. MCI mild cognitive impairment.

As shown in Fig. 2, the pooled prevalence of MCI based on the 53 included studies was 21.2% (95% CI: 18.7–23.6%; *I*^2^ = 99.6%).

### Subgroup and meta-regression analyses

Table 2 presents the results of subgroup analyses. The screening tools used for MCI (Q = 16.51, *P* = 0.011) were significantly associated with the prevalence of MCI. Studies using the Montreal Cognitive Assessment (MoCA) (49.8%, 95% CI: 0–123.4%) had a higher prevalence of MCI than those using other instruments. For the meta-regression analyses, there were no significant associations between the prevalence of MCI and mean age (*t* = 0.54, *P* = 0.591), male proportion (*t* = −0.97, *P* = 0.340), and quality assessment score (*t* = 0.13, *P* = 0.900; Figs. S1–3).

**Table 2:** Subgroup analysis of the prevalence of mild cognitive impairment.

Subgroups	Categories	No. of studies	Event	Total	Prevalence (%)	95% CI (%)	*I*^2^ (%)	*P* value within subgroup	*Q* (*P* value across subgroups)
Region	Europe & Central Asia	29	12,867	55,259	19.7	14.9–24.5	99.5	<0.001	0.35 (0.840)
	North America	14	69,857	315,901	20.7	16.7–24.8	99.8	<0.001	–
	East Asia & Pacific	8	762	4557	18.2	10.7–25.8	98.1	<0.001	–
Countries by income	Upper middle income	8	885	4631	26.4	12.7–40.0	99.5	<0.001	0.75 (0.388)
	High income	45	82,798	371,408	20.3	17.7–22.8	99.6	<0.001	–
Sample method	Random	16	4144	18,926	19.3	15.8–22.7	96.9	<0.001	3.83 (0.147)
	Convenience	31	77,684	347,134	23.4	20.1–26.8	99.8	<0.001	–
	Cluster	3	1289	6764	14.7	1.4–28.0	99.5	<0.001	–
Screening scale	CAREDiag	2	478	2166	28.7	12.5–44.9	94.2	<0.001	16.51 (0.011)
	CPS	9	23,265	149,679	14.7	13.0–16.3	97.1	<0.001	–
	GCS	4	7173	20,337	32.4	15.5–49.4	99.9	<0.001	–
	MMSE	25	3216	16,897	19.5	15.7–23.4	98.0	<0.001	–
	MoCA	2	229	751	49.8	0–123.4	99.9	<0.001	–
	Pfeiffer test	3	117	599	18.8	6.8–30.8	93.7	<0.001	–
	SPMSQ	3	116	1182	9.6	3.6–15.6	90.5	<0.001	–
Age group (years)	70–74	2	42	581	18.2	0–45.1	90.1	0.001	2.84 (0.417)
	75–79	7	524	3587	15.0	7.4–22.6	97.8	<0.001	–
	80–84	16	2645	13,633	21.7	17.0–26.4	98.2	<0.001	–
	85–89	15	24,494	146,490	17.9	15.6–20.2	98.1	<0.001	–
Survey starting year	Before 2000	4	7082	25,816	34.9	9.5–60.2	99.9	<0.001	4.85 (0.304)
	2000–2004	2	2915	17,132	11.6	0.30–23.0	99.2	<0.001	–
	2005–2009	9	19,126	120,602	17.0	13.3–20.8	98.1	<0.001	–
	2010–2014	12	51,085	194,489	19.9	17.2–22.5	96.4	<0.001	–
	2015–2019	7	873	5600	16.8	10.0–23.5	98.3	<0.001	–

*CAREDiag* The Care Dementia Diagnostic Scale, *CPS* Minimum Data Set 2.0-Cognitive Performance Scale, *GCS* Gottfries cognitive scale, *MMSE* Mini-Mental State Examination, *MoCA* Montreal Cognitive Assessment, *SPSMQ* Short Portable Mental Status Questionnaire.

### Sensitivity analysis and publication bias

**Fig. 3:**
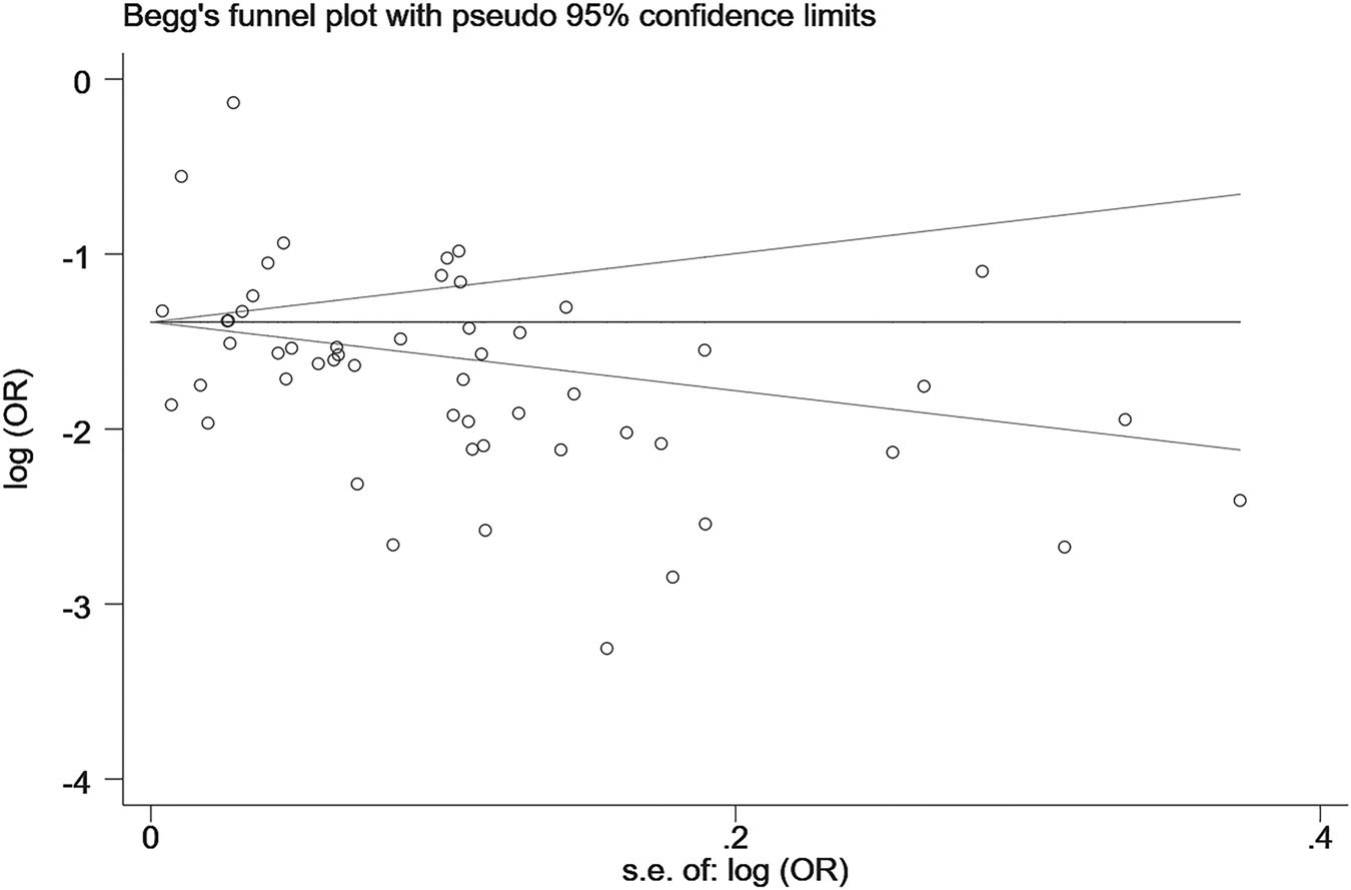
Publication bias of included studies on the prevalence of MCI. Funnel plot was plotted by random effects. MCI mild cognitive impairment.

The results of sensitivity analyses are shown in Fig. S4. We did not find any outlying studies that could significantly affect the primary results. The Begg’s funnel plot (Begg’s test: *z* = −0.92, *P* = 0.357) and Egger’s test (*t* = −0.93; *P* = 0.358) did not find any significant publication biases (Fig. 3).

## Discussion

To the best of our knowledge, this was the first meta-analysis to estimate the pooled global prevalence of MCI among older adults living in nursing homes. This meta-analysis included 53 studies across 17 countries and found an overall prevalence of 21.2% (95% CI: 18.7–23.6%; *I*^2^ = 99.6%), which is higher than the findings in the general community populations (17.3%; 95% CI: 13.8–20.8%) [24] and Chinese community-dwelling populations (12.2%; 95% CI: 10.6–14.2%) [22]. There are several reasons for this discrepancy. Cognitive impairment is one of the major reasons for admission to nursing homes [25, 38], which would result in a higher rate of MCI in nursing homes residents compared to community-dwelling population. In addition, other key reasons for nursing home placement include major physical and psychiatric disorders, such as physical pain [39], diabetes [40], depression [41], and anxiety [42], all of which could be associated with a higher risk of cognitive impairment [43, 44].

MCI is a pathological condition that encompasses a series of symptoms related to cognition rather than being defined as a disease [45]. It often evolves gradually into memory loss and difficulty in communication and handling complex tasks, visual and spatial abilities, planning and organization, coordination and motor functions, and disorientation [46]. Thus, early identification is crucial to prevent the deterioration of cognition impairment [4]. Despite the high conversion rate to dementia, there remains a small proportion of persons with MCI who can recover to a normal cognitive level [10], which highlights the importance of early management of MCI. Certain interventional measures for MCI, such as cognitive training [47], physical exercise [48], and diet regulation [48], appeared to have some symptomatic benefits although there is no effective pharmacological treatment for MCI [45, 49]. The guidelines for the management of MCI propose a multi-targeted treatment approach [45], which includes a range of strategies to improve cognitive performance in this population.

Various scales are used to screen MCI such as the MoCA, Mini-Mental State Examination (MMSE), Cognitive Performance Scale (CPS), Gottfries cognitive scale (GCS), Pfeiffer test,) and Short Portable Mental Status Questionnaire (SPSMQ). In this study, subgroup analyses revealed a significant difference in MCI prevalence between different MCI screening tools used, with those using the MoCA having the highest prevalence (49.8%). MoCA is a brief cognitive screening tool with excellent sensitivity (90%) and specificity (87%) [50, 51], which covers short-term memory, visuospatial skills, executive function, attention, concentration and working memory, language, and orientation. The MoCA assessment requires 10–20 min to complete, which is influenced by the education level of the participant, hence, an extra point is added to the MoCA total score for participants with <13 years of education [52]. The MMSE is another widely used tool in screening cognition levels, with acceptable sensitivity (13–97%) and specificity (60–100%) across different studies [53–55], that covers multiple domains, including orientation, attention and calculation, language, immediate recall, short-term memory, registration, and construct ability. The MMSE assessment needs 5–10 min to complete, which is also associated with the education level of the patient [52]. However, the MMSE is not a reliable test for detecting MCI at an early stage [53]. Sensitivity, specificity, and time efficiency are the main factors in evaluating such screening tools [52], therefore, various screening tools could contribute to different results for MCI prevalence [56]. Previous studies found that the MoCA showed better specificity and sensitivity in detecting MCI than other cognitive measures such as the MMSE [50, 53, 54]. However, it should be noted that as only two studies using the MoCA were included in our meta-analysis, this finding may be preliminary and needs to be confirmed in future studies. The CPS, which is similar to the MMSE in identifying cognitive impairment, was initially applied to nursing home residents with good sensitivity (87–94%) and specificity (80–95%) [57]. Although the CPS assessment is not influenced by age and education level, it requires more than 30 min for completion [52]. Overall, the assessment duration and the potential influence of education level on the results should be considered in selecting a suitable screening instrument for MCI.

Older age is a risk factor for cognitive decline and could accelerate the progression of cognitive impairment [56, 58]. The findings on the relationship between age and MCI prevalence were mixed. Some studies did not find a significant association [8, 59], while others showed a significant association between age and MCI prevalence, with a higher prevalence in older individuals [56, 60–62]. In this meta-analysis, the pooled prevalence of MCI was 18.2%, 15.0%, and 21.7% in the 70–74-, 75–79-, and 80–84-years age groups, respectively, but the difference between age groups did not reach a significant level.

Similarly, the association between gender and MCI prevalence is also controversial. For instance, some studies did not find a significant association between gender and MCI prevalence [56, 60, 62], while other studies reported that either males [8, 61] or females had a higher prevalence of MCI [59]. One study attributed the possible reason for higher MCI prevalence in males to the higher proportion of males in the study sample [8]. The higher prevalence of MCI in females may be due to hormonal differences between males and females [22]; i.e., estrogen exposure plays a role in brain aging, which is associated with changes of global cognitive functioning and verbal attention [63]. The decreased estrogen levels in females after menopause can lead to partial impairment of cognitive function such as verbal memory, reasoning, and vigilance [64]. In this meta-analysis, however, there were no significant gender difference in terms of MCI prevalence.

We also did not find significant differences in the prevalence of MCI among older adults in nursing homes between geographical regions and between different income levels, which are not consistent with the findings in the community-dwelling older populations [65]. The pooled prevalence of MCI among older people living in nursing homes was 19.7% in Europe & Central Asia, 20.7% in North America, and 18.2% in East Asia & Pacific in this study, while the corresponding rate was 10.9%, 15.5% and 19.0%, respectively among community-dwelling older populations [65]. We speculate that compared to those living in the community, older adults living in nursing homes usually received better support and health care, which could offset the differences of MCI prevalence caused by different regions and economic factors.

The strengths of this meta-analysis included a large number of studies, the use of sophisticated analyses (e.g., subgroup and meta-regression analyses) and the homogeneous study sample of nursing homes residents. However, several limitations warrant attention in this study. Firstly, significant heterogeneity between studies remained even when subgroup analyses were performed, since heterogeneity is a common phenomenon in the meta-analysis of epidemiological surveys [66]. Second, some factors associated with the prevalence of MCI, such as education level, economic status, marital status, and MCI subtypes, were not examined due to insufficient data. Third, MCI was assessed using self-report scales in most studies, rather than diagnostic clinical interviews. MCI prevalence among those living in nursing homes may be better examined based on standard diagnostic criteria such as the Petersen’s criteria [2].

In summary, this meta-analysis showed that the global prevalence of MCI was over 20% among older adults living in nursing homes. Adequate screening measures and allocation of resources are needed to address the high global prevalence of MCI among older adults living in nursing homes. Early identification, preventive interventions and dementia treatment and care are essential to reduce the health burden of MCI in this population.

## Supplementary information

Supplementary material
