# Correction to: Global prevalence of mild cognitive impairment among older adults living in nursing homes: a meta-analysis and systematic review of epidemiological surveys

**DOI:** 10.1038/s41398-023-02408-3

**Published:** 2023-03-30

**Authors:** Pan Chen, Hong Cai, Wei Bai, Zhaohui Su, Yi-Lang Tang, Gabor S. Ungvari, Chee H. Ng, Qinge Zhang, Yu-Tao Xiang

**Affiliations:** 1 Unit of Psychiatry, Department of Public Health and Medicinal Administration, & Institute of Translational Medicine, Faculty of Health Sciences, University of Macau, Macao, Macao SAR China; 2 Centre for Cognitive and Brain Sciences, University of Macau, Macao, Macao SAR China; 3 Institute of Advanced Studies in Humanities and Social Sciences, University of Macau, Macao, SAR China; 4 School of Public Health, Southeast University, Nanjing, China; 5 Department of Psychiatry and Behavioral Sciences, Emory University, Atlanta, GA USA; 6 Atlanta Veterans Affairs Medical Center, Decatur, GA USA; 7 Section of Psychiatry, University of Notre Dame Australia, Fremantle, Australia; 8 Division of Psychiatry, School of Medicine, University of Western Australia, Perth, Australia; 9 Department of Psychiatry, The Melbourne Clinic and St Vincent’s Hospital, University of Melbourne, Richmond, Victoria, Australia; 10 Beijing Key Laboratory of Mental Disorders, National Clinical Research Center for Mental Disorders, National Center for Mental Disorders, Beijing Anding Hospital, Capital Medical University & Advanced Innovation Center for Human Brain Protection, Capital Medical University, Beijing, China

**Keywords:** Psychiatric disorders, Scientific community

Correction to: *Translational Psychiatry* 10.1038/s41398-023-02361-1, published online 11 March 2023

The original version of this article contained errors in the authorship and the affiliations. The correct authorship and affiliations can be found above. The original article has been corrected.

