# Supplementary material for: Global prevalence of mild cognitive impairment among older adults living in nursing homes: a meta-analysis and systematic review of epidemiological surveys
Source: Transl Psychiatry. 2023 Mar 11;13:88. doi: 10.1038/s41398-023-02361-1 (PMC10008549; doi:10.1038/s41398-023-02361-1)
Supplement: Supplementary file 1 — Supplementary material [file 41398_2023_2361_MOESM1_ESM.docx]

**Supplementary materials**

Figure S1. Meta-regression analysis of mean age on the prevalence of mild cognitive impairment

Figure S2. Meta-regression analysis of male gender on prevalence of mild cognitive impairment

Figure S3. Meta-regression analysis of study quality assessment on the prevalence of mild cognitive impairment

Figure S4. Sensitivity analysis

Figure S1. Meta regression analysis of mean age on the prevalence of mild cognitive impairment

**
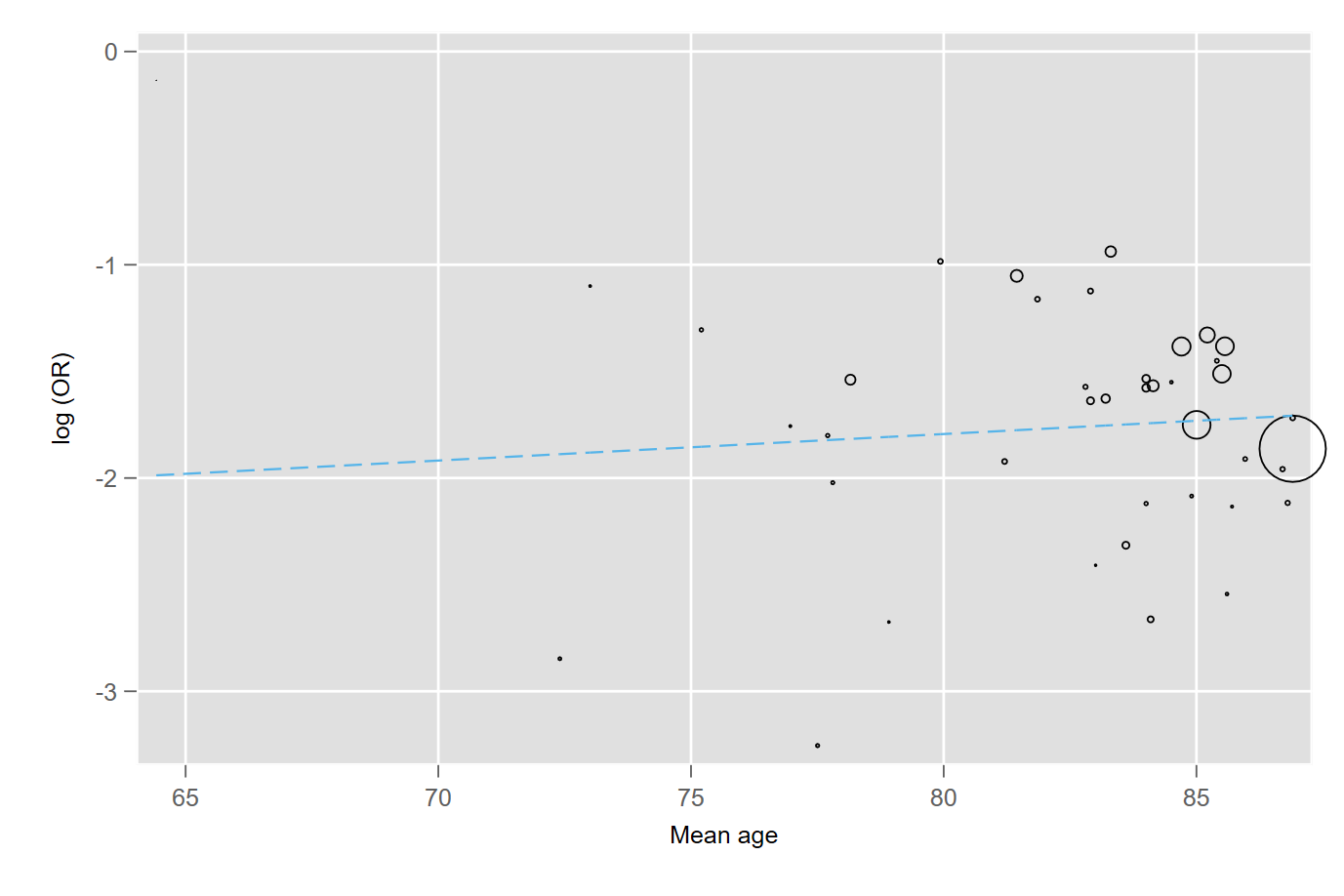
**

Coefficient=0.012, Standard error=0.023, t=0.54, P=0.591

Figure S2. Meta-regression analysis of male gender on prevalence of mild cognitive impairment


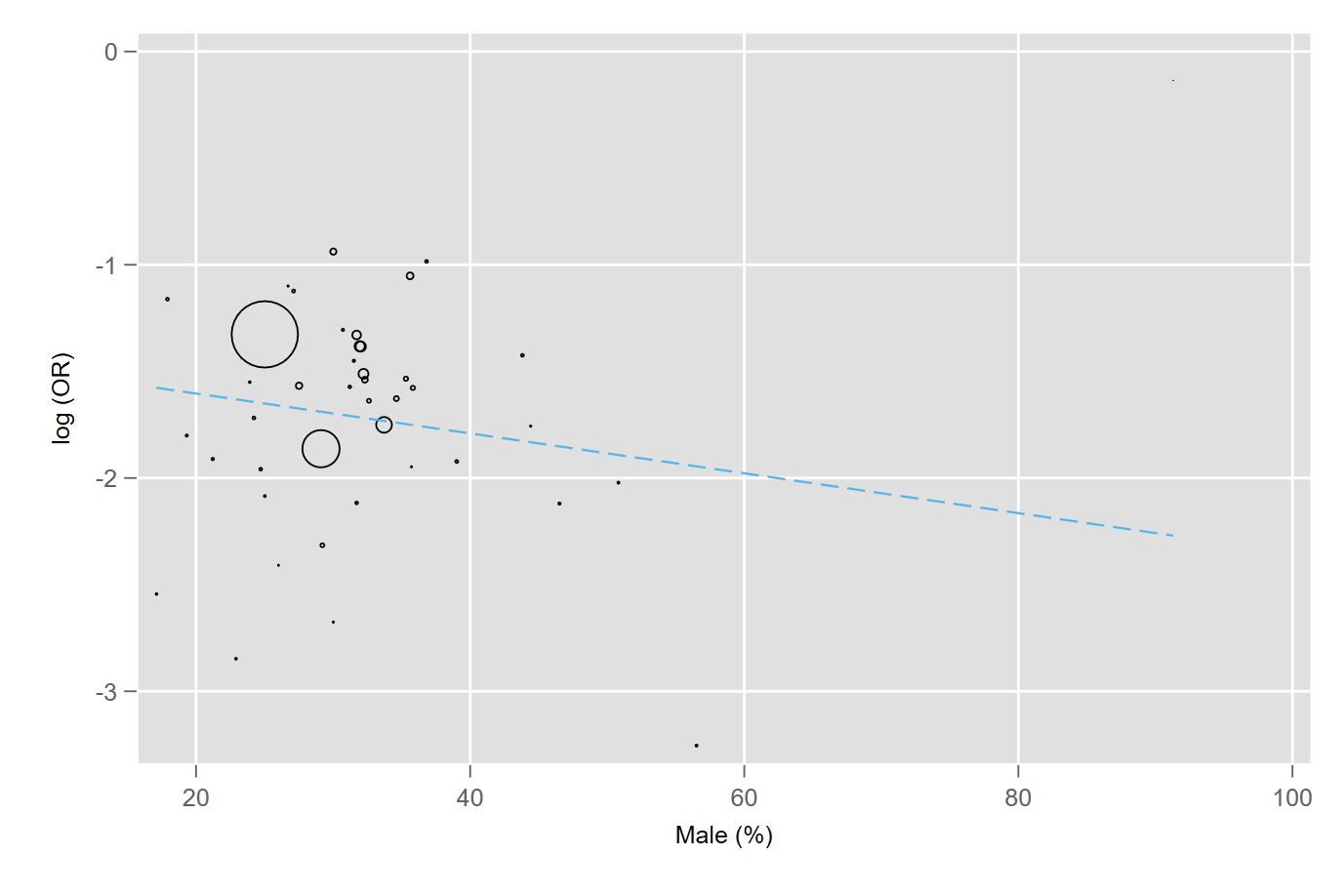
Coefficient=-0.009, Standard error=0.010, t=-0.97, P=0.340

Figure S3. Meta regression analysis of study quality assessment on the prevalence of mild cognitive impairment


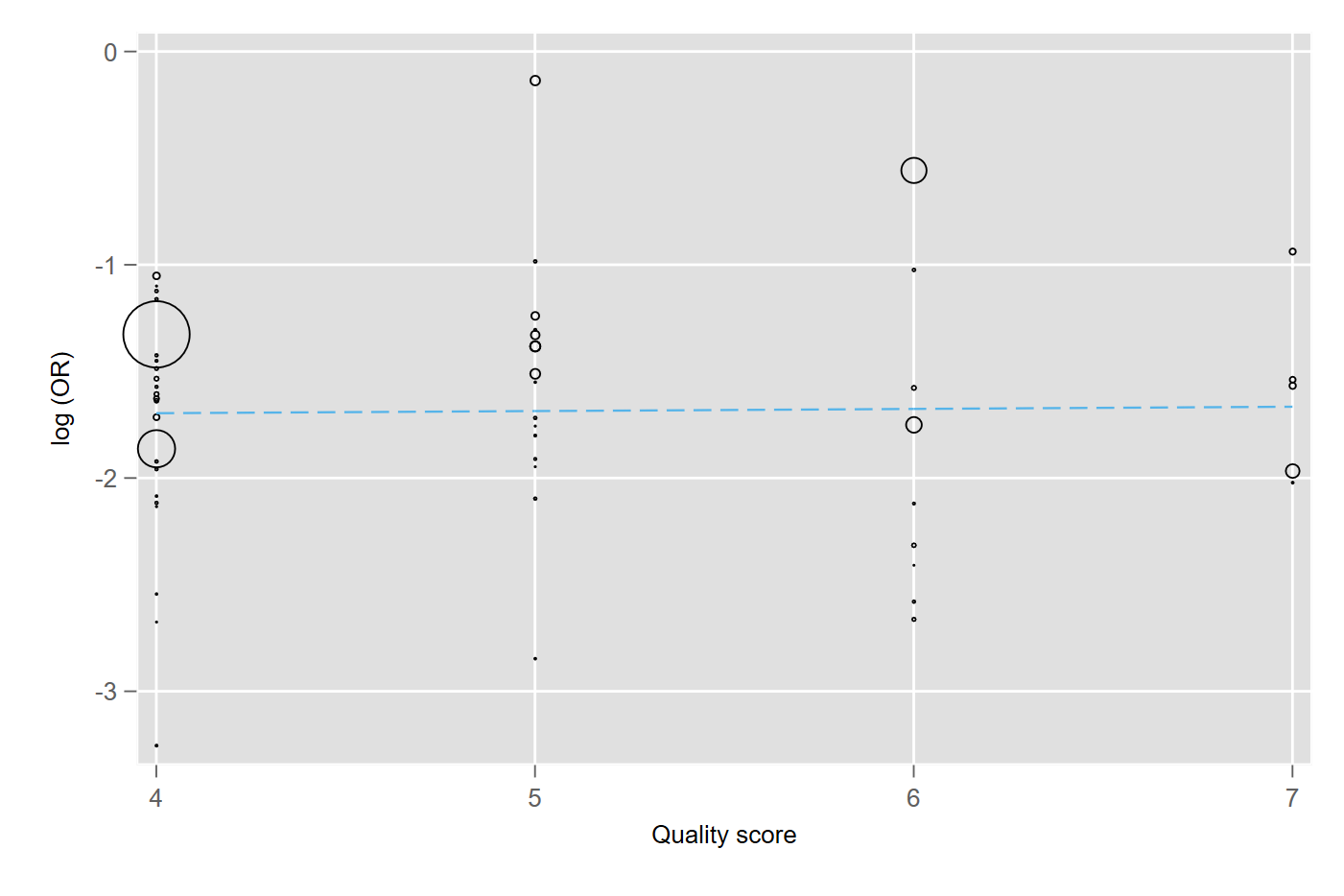


Coefficient=0.010, Standard error=0.080, t=0.13, P=0.900

Figure S4. Sensitivity analysis

**
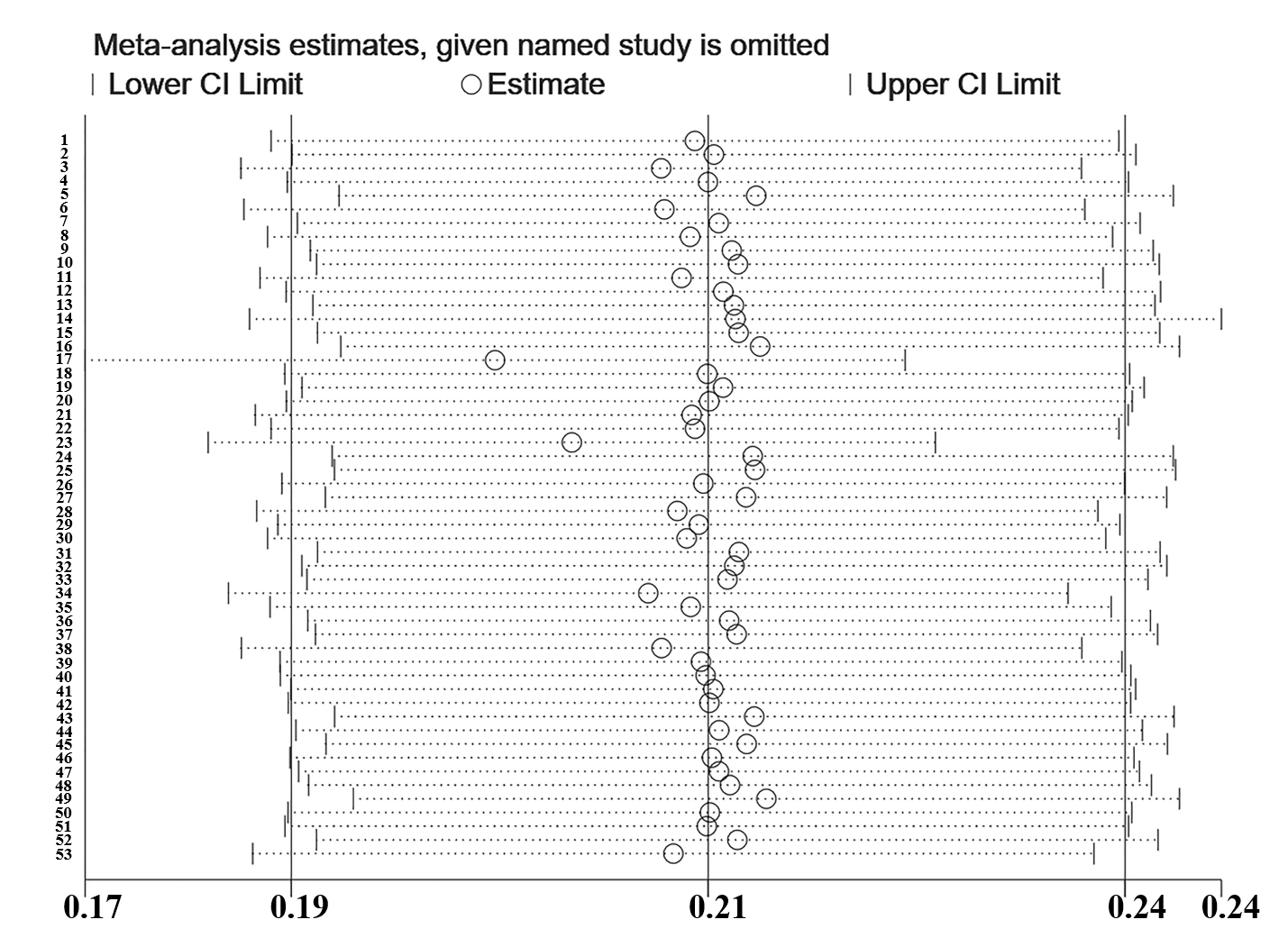
**

Table S1. Search strategy

| Databases | Keywords | Search terms |
| --- | --- | --- |
| PubMed | #1= ("cognitive dysfunction"[MeSH Terms] OR "mild cognitive impairment" or "MCI")  #2= ("Nursing Homes" OR "Nursing Home" OR “Intermediate Care Facilities” OR “Intermediate Care Facility” OR “Skilled Nursing Facilities” OR “Skilled Nursing Facility” OR “Extended Care Facilities” OR “Extended Care Facility” OR “convalescence home” OR “convalescence hospital” OR “long-term care” OR “old age homes” OR “residential homes” OR “nursing home*” OR “residential care” OR “institutionalization*” OR “nursing home placement*” OR “nursing home admission*” OR “Homes, Nursing”)  #3= ("aged" OR "old age" OR "elderly" OR "late-life" OR “geriatric*” OR “older adult” OR “elder*”)  #4= ("prevalence" OR "epidemiology" OR “rate”) | #1 AND #2 AND #3 AND #4 |
| Web of Science  PsycINFO  Embase  CINAHL Complete | #1= ("cognitive dysfunction" OR "mild cognitive impairment" or "MCI")  #2= ("Nursing Homes" OR "Nursing Home" OR “Intermediate Care Facilities” OR “Intermediate Care Facility” OR “Skilled Nursing Facilities” OR “Skilled Nursing Facility” OR “Extended Care Facilities” OR “Extended Care Facility” OR “convalescence home” OR “convalescence hospital” OR “long-term care” OR “old age homes” OR “residential homes” OR “nursing home*” OR “residential care” OR “institutionalization*” OR “nursing home placement*” OR “nursing home admission*” OR “Homes, Nursing”)  #3= ("aged" OR "old age" OR "elderly" OR "late-life" OR “geriatric*” OR “older adult” OR “elder*”)  #4= ("prevalence" OR "epidemiology" OR “rate”) | #1 AND #2 AND #3 AND #4 |

Table S2. Quality assessment of included studies

| **NO.** | **Study** | **1. Target population was defined clearly** | **2. Probability sampling or entire population surveyed** | **3. Response rate was equal or greater than 80%** | **4. Non-responders were clearly described** | **5. Sample was representative of the target population** | **6. Data collection methods was standardized** | **7. Validated criteria was used to diagnose MDD** | **8. Prevalence estimates were given with confidence intervals and detailed by subgroups (if applicable)** | **Total score** |
| --- | --- | --- | --- | --- | --- | --- | --- | --- | --- | --- |
| 1 | Björk et al. 2018 [59] | 1 | 1 | 0 | 0 | 1 | 1 | 1 | 0 | 5 |
| 2 | Bo et al. 2015 [40] | 1 | 0 | 0 | 0 | 1 | 1 | 1 | 0 | 4 |
| 3 | Chun et al. 2017 [67] | 1 | 0 | 0 | 1 | 1 | 1 | 1 | 0 | 5 |
| 4 | Closs et al. 2004 [68] | 1 | 0 | 0 | 1 | 1 | 1 | 1 | 0 | 5 |
| 5 | Cocco et al. 2018 [69] | 1 | 0 | 1 | 0 | 1 | 1 | 1 | 0 | 6 |
| 6 | Creighton et al. 2019 [70] | 1 | 1 | 0 | 1 | 1 | 1 | 1 | 0 | 6 |
| 7 | de Jong-Schmit et al. 2021 [71] | 1 | 1 | 0 | 0 | 1 | 1 | 1 | 0 | 5 |
| 8 | Díaz et al. 2020 [72] | 1 | 1 | 0 | 0 | 1 | 1 | 1 | 0 | 5 |
| 9 | Garcia-Gollarte et al. 2020 [73] | 1 | 0 | 0 | 0 | 1 | 1 | 1 | 0 | 4 |
| 10 | Gjøra et al. 2021 [61] | 1 | 0 | 0 | 1 | 1 | 1 | 1 | 0 | 5 |
| 11 | Gruber-Baldini et al. 2004 [74] | 1 | 1 | 0 | 0 | 1 | 1 | 1 | 0 | 5 |
| 12 | Guliani et al. 2021 [75] | 1 | 0 | 1 | 1 | 1 | 1 | 1 | 0 | 6 |
| 13 | Guo et al. 2012 [8] | 1 | 1 | 1 | 1 | 1 | 1 | 1 | 0 | 7 |
| 14 | Guthrie et al. 2018 [76] | 1 | 0 | 0 | 0 | 1 | 1 | 1 | 0 | 4 |
| 15 | Hagglund et al. 2019 [77] | 1 | 1 | 0 | 1 | 1 | 1 | 1 | 0 | 6 |
| 16 | Hasche et al. 2010 [78] | 1 | 1 | 0 | 0 | 1 | 1 | 1 | 0 | 5 |
| 17 | Hayajneh et al. 2020 [18] | 1 | 1 | 0 | 0 | 1 | 1 | 1 | 0 | 5 |
| 18 | Kijowska et al. 2018 [25] | 1 | 1 | 1 | 1 | 1 | 1 | 1 | 0 | 7 |
| 19 | Kowalska et al. 2013 [79] | 1 | 0 | 0 | 1 | 1 | 1 | 1 | 0 | 5 |
| 20 | Lachs et al. 2016 [80] | 1 | 1 | 1 | 1 | 1 | 1 | 1 | 0 | 7 |
| 21 | Lapane et al. 2020 [81] | 1 | 0 | 0 | 0 | 1 | 1 | 1 | 0 | 4 |
| 22 | Lindbo et al. 2017 [82] | 1 | 0 | 0 | 1 | 1 | 1 | 1 | 0 | 5 |
| 23 | Lövheim et al. 2010 [83] | 1 | 1 | 0 | 1 | 1 | 1 | 1 | 0 | 6 |
| 24 | Lueken et al. 2007 [84] | 1 | 0 | 0 | 0 | 1 | 1 | 1 | 0 | 4 |
| 25 | Malara et al. 2014 [85] | 1 | 0 | 0 | 0 | 1 | 1 | 1 | 0 | 4 |
| 26 | Mansbach et al. 2016 [26] | 1 | 0 | 0 | 0 | 1 | 1 | 1 | 0 | 4 |
| 27 | Manz et al. 2000 [86] | 1 | 1 | 0 | 1 | 1 | 1 | 1 | 0 | 6 |
| 28 | Margari et al. 2012 [87] | 1 | 0 | 0 | 0 | 1 | 1 | 1 | 0 | 4 |
| 29 | McCusker et al. 2014 [41] | 1 | 0 | 0 | 0 | 1 | 1 | 1 | 0 | 4 |
| 30 | McDougall et al. 2001 [88] | 1 | 0 | 0 | 0 | 1 | 1 | 1 | 0 | 4 |
| 31 | Namasivayam-MacDonald et al. 2018 [89] | 1 | 0 | 0 | 0 | 1 | 1 | 1 | 0 | 4 |
| 32 | Netten et al. 2001 [90] | 1 | 0 | 1 | 1 | 1 | 1 | 1 | 0 | 7 |
| 33 | Onishi et al. 2013 [91] | 1 | 1 | 0 | 0 | 1 | 1 | 1 | 0 | 5 |
| 34 | Parmelee et al. 1993 [92] | 1 | 1 | 1 | 1 | 1 | 1 | 1 | 0 | 7 |
| 35 | Ramlall et al. 2013 [56] | 1 | 1 | 0 | 1 | 1 | 1 | 1 | 0 | 5 |
| 36 | Redaelli et al. 2020 [93] | 1 | 0 | 0 | 0 | 1 | 1 | 1 | 0 | 5 |
| 37 | Rodríguez-Rejón et al. 2020 [94] | 1 | 0 | 0 | 0 | 1 | 1 | 1 | 0 | 4 |
| 38 | Seijo-Martinez et al. 2016 [95] | 1 | 0 | 0 | 0 | 1 | 1 | 1 | 0 | 4 |
| 39 | Sjölund et al. 2021 [96] | 1 | 0 | 0 | 0 | 1 | 1 | 1 | 0 | 4 |
| 40 | Skoldunger et al. 2019 [97] | 1 | 1 | 0 | 0 | 1 | 1 | 1 | 0 | 5 |
| 41 | Steenbeek et al. 2021 [98] | 1 | 0 | 0 | 0 | 1 | 1 | 1 | 0 | 4 |
| 42 | Sutcliffe et al. 2007 [99] | 1 | 0 | 0 | 0 | 1 | 1 | 1 | 0 | 4 |
| 43 | Thompson et al. 2017 [100] | 1 | 1 | 0 | 1 | 1 | 1 | 1 | 0 | 6 |
| 44 | Vincze et al. 2007 [62] | 1 | 0 | 0 | 0 | 1 | 1 | 1 | 0 | 4 |
| 45 | Volicer et al. 2011 [101] | 1 | 0 | 1 | 1 | 1 | 1 | 1 | 0 | 6 |
| 46 | Wang et al. 2020 [102] | 1 | 0 | 0 | 0 | 1 | 1 | 1 | 0 | 4 |
| 47 | Wongpakaran et al. 2012 [103] | 1 | 0 | 1 | 0 | 1 | 1 | 1 | 0 | 5 |
| 48 | Wulff et al. 2013 [104] | 1 | 0 | 0 | 0 | 1 | 1 | 1 | 0 | 4 |
| 49 | Xu et al. 2019 [20] | 1 | 0 | 0 | 0 | 1 | 1 | 1 | 0 | 4 |
| 50 | Xu et al. 2017 [105] | 1 | 0 | 1 | 0 | 1 | 1 | 1 | 0 | 6 |
| 51 | Yang et al. 2019 [60] | 1 | 0 | 0 | 0 | 1 | 1 | 1 | 0 | 4 |
| 52 | Zuluaga et al. 2012a [106] | 1 | 0 | 0 | 0 | 1 | 1 | 1 | 0 | 4 |
| 53 | Zuluaga et al. 2012b [107] | 1 | 0 | 0 | 0 | 1 | 1 | 1 | 0 | 4 |

Table S3. PRISMA 2020 Checklist

| **Section and Topic** | **Item #** | **Checklist item** | **Location where item is reported** |
| --- | --- | --- | --- |
| **TITLE** | | |  |
| Title | 1 | Identify the report as a systematic review. | Title |
| **ABSTRACT** | | |  |
| Abstract | 2 | See the PRISMA 2020 for Abstracts checklist. | Abstract (Checklist See Table S4) |
| **INTRODUCTION** | | |  |
| Rationale | 3 | Describe the rationale for the review in the context of existing knowledge. | Introduction |
| Objectives | 4 | Provide an explicit statement of the objective(s) or question(s) the review addresses. | Introduction |
| **METHODS** | | |  |
| Eligibility criteria | 5 | Specify the inclusion and exclusion criteria for the review and how studies were grouped for the syntheses. | Methods: inclusion and exclusion criteria |
| Information sources | 6 | Specify all databases, registers, websites, organisations, reference lists and other sources searched or consulted to identify studies. Specify the date when each source was last searched or consulted. | Methods: Search strategy |
| Search strategy | 7 | Present the full search strategies for all databases, registers and websites, including any filters and limits used. | Methods: Search strategy |
| Selection process | 8 | Specify the methods used to decide whether a study met the inclusion criteria of the review, including how many reviewers screened each record and each report retrieved, whether they worked independently, and if applicable, details of automation tools used in the process. | Methods: Search strategy, inclusion and exclusion criteria |
| Data collection process | 9 | Specify the methods used to collect data from reports, including how many reviewers collected data from each report, whether they worked independently, any processes for obtaining or confirming data from study investigators, and if applicable, details of automation tools used in the process. | Methods: Data extraction and study quality assessment |
| Data items | 10a | List and define all outcomes for which data were sought. Specify whether all results that were compatible with each outcome domain in each study were sought (e.g. for all measures, time points, analyses), and if not, the methods used to decide which results to collect. | Methods: Data extraction and study quality assessment |
|  | 10b | List and define all other variables for which data were sought (e.g. participant and intervention characteristics, funding sources). Describe any assumptions made about any missing or unclear information. | Methods: Data extraction and study quality assessment |
| Study risk of bias assessment | 11 | Specify the methods used to assess risk of bias in the included studies, including details of the tool(s) used, how many reviewers assessed each study and whether they worked independently, and if applicable, details of automation tools used in the process. | Methods: Data extraction and study quality assessment |
| Effect measures | 12 | Specify for each outcome the effect measure(s) (e.g. risk ratio, mean difference) used in the synthesis or presentation of results. | Methods: Statistical analysis |
| Synthesis methods | 13a | Describe the processes used to decide which studies were eligible for each synthesis (e.g. tabulating the study intervention characteristics and comparing against the planned groups for each synthesis (item #5)). | Methods: Search strategy, Statistical analysis |
|  | 13b | Describe any methods required to prepare the data for presentation or synthesis, such as handling of missing summary statistics, or data conversions. | Methods: Statistical analysis |
|  | 13c | Describe any methods used to tabulate or visually display results of individual studies and syntheses. | Methods: Statistical analysis |
|  | 13d | Describe any methods used to synthesize results and provide a rationale for the choice(s). If meta-analysis was performed, describe the model(s), method(s) to identify the presence and extent of statistical heterogeneity, and software package(s) used. | Methods: Statistical analysis |
|  | 13e | Describe any methods used to explore possible causes of heterogeneity among study results (e.g. subgroup analysis, meta-regression). | Methods: Statistical analysis |
|  | 13f | Describe any sensitivity analyses conducted to assess robustness of the synthesized results. | Methods: Statistical analysis |
| Reporting bias assessment | 14 | Describe any methods used to assess risk of bias due to missing results in a synthesis (arising from reporting biases). | Methods: Statistical analysis |
| Certainty assessment | 15 | Describe any methods used to assess certainty (or confidence) in the body of evidence for an outcome. | Methods: Statistical analysis |
| **RESULTS** | | |  |
| Study selection | 16a | Describe the results of the search and selection process, from the number of records identified in the search to the number of studies included in the review, ideally using a flow diagram. | Result: Characteristics of the studies, Figure 1 |
|  | 16b | Cite studies that might appear to meet the inclusion criteria, but which were excluded, and explain why they were excluded. | Result: Characteristics of the studies, Figure 1 |
| Study characteristics | 17 | Cite each included study and present its characteristics. | Result: Characteristics of the studies, Table 1 |
| Risk of bias in studies | 18 | Present assessments of risk of bias for each included study. | Result: Characteristics of the studies, Table 1 |
| Results of individual studies | 19 | For all outcomes, present, for each study: (a) summary statistics for each group (where appropriate) and (b) an effect estimate and its precision (e.g. confidence/credible interval), ideally using structured tables or plots. | Result: Characteristics of the studies, Table 1 |
| Results of syntheses | 20a | For each synthesis, briefly summarise the characteristics and risk of bias among contributing studies. | Result: Characteristics of the studies, Table 1 |
|  | 20b | Present results of all statistical syntheses conducted. If meta-analysis was done, present for each the summary estimate and its precision (e.g. confidence/credible interval) and measures of statistical heterogeneity. If comparing groups, describe the direction of the effect. | Result: Prevalence of mild cognitive impairment, subgroup and meta-regression analyses |
|  | 20c | Present results of all investigations of possible causes of heterogeneity among study results. | Result: Prevalence of mild cognitive impairment, subgroup and meta-regression analyses, Table 2, Figure S1-3 |
|  | 20d | Present results of all sensitivity analyses conducted to assess the robustness of the synthesized results. | Result: Sensitivity analysis and publication bias, Figure 3 |
| Reporting biases | 21 | Present assessments of risk of bias due to missing results (arising from reporting biases) for each synthesis assessed. | Result: Sensitivity analysis and publication bias, Figure 3 |
| Certainty of evidence | 22 | Present assessments of certainty (or confidence) in the body of evidence for each outcome assessed. | Result: Sensitivity analysis and publication bias, Figure 3 |
| **DISCUSSION** | | |  |
| Discussion | 23a | Provide a general interpretation of the results in the context of other evidence. | Discussion: First paragraph |
|  | 23b | Discuss any limitations of the evidence included in the review. | Discussion: Seventh paragraph |
|  | 23c | Discuss any limitations of the review processes used. | Discussion: Seventh paragraph |
|  | 23d | Discuss implications of the results for practice, policy, and future research. | Discussion: Last paragraph |
| **OTHER INFORMATION** | | |  |
| Registration and protocol | 24a | Provide registration information for the review, including register name and registration number, or state that the review was not registered. | Methods: Search strategy |
|  | 24b | Indicate where the review protocol can be accessed, or state that a protocol was not prepared. | Methods: Search strategy |
|  | 24c | Describe and explain any amendments to information provided at registration or in the protocol. | NA |
| Support | 25 | Describe sources of financial or non-financial support for the review, and the role of the funders or sponsors in the review. | Additional information |
| Competing interests | 26 | Declare any competing interests of review authors. | Additional information |
| Availability of data, code and other materials | 27 | Report which of the following are publicly available and where they can be found: template data collection forms; data extracted from included studies; data used for all analyses; analytic code; any other materials used in the review. | Additional information |

Table S4. PRISMA 2020 Abstract Checklist

| **Section and Topic** | **Item #** | **Checklist item** | **Reported (Yes/No)** |
| --- | --- | --- | --- |
| **TITLE** | | |  |
| Title | 1 | Identify the report as a systematic review. | Yes |
| **BACKGROUND** | | |  |
| Objectives | 2 | Provide an explicit statement of the main objective(s) or question(s) the review addresses. | Yes |
| **METHODS** | | |  |
| Eligibility criteria | 3 | Specify the inclusion and exclusion criteria for the review. | Yes |
| Information sources | 4 | Specify the information sources (e.g. databases, registers) used to identify studies and the date when each was last searched. | Yes |
| Risk of bias | 5 | Specify the methods used to assess risk of bias in the included studies. | Yes |
| Synthesis of results | 6 | Specify the methods used to present and synthesise results. | Yes |
| **RESULTS** | | |  |
| Included studies | 7 | Give the total number of included studies and participants and summarise relevant characteristics of studies. | Yes |
| Synthesis of results | 8 | Present results for main outcomes, preferably indicating the number of included studies and participants for each. If meta-analysis was done, report the summary estimate and confidence/credible interval. If comparing groups, indicate the direction of the effect (i.e. which group is favoured). | Yes |
| **DISCUSSION** | | |  |
| Limitations of evidence | 9 | Provide a brief summary of the limitations of the evidence included in the review (e.g. study risk of bias, inconsistency and imprecision). | Yes |
| Interpretation | 10 | Provide a general interpretation of the results and important implications. | Yes |
| **OTHER** | | |  |
| Funding | 11 | Specify the primary source of funding for the review. | No |
| Registration | 12 | Provide the register name and registration number. | Yes |
